# High-Mannose *N*‑Glycans To Monitor Early Response to Chemotherapy in African Epithelial Ovarian Cancer Patients—A Pilot Study

**DOI:** 10.1021/acs.jproteome.5c00442

**Published:** 2025-12-10

**Authors:** Francis M. Wanyama, Obinna Umeh, Karina Biskup, Rudolf Tauber, Alfred Mokomba, Catherine Nyongesa, Véronique Blanchard

**Affiliations:** † Institute of Diagnostic Laboratory Medicine, Clinical Chemistry and Pathobiochemistry, 14903 Charité-Universitätsmedizin Berlin, corporate member of Freie Universität Berlin, Humboldt-Universität zu Berlin and Berlin Institute of Health. Post: Augustenburger Platz 1, Berlin D-13353, Germany; ‡ Department of Human Pathology, Thematic Unit of Clinical Chemistry, 108330 University of Nairobi, P.O. Box 19676, Nairobi 00202, Kenya; § Department of Human Medicine, Medical School Berlin, Rüdesheimer Straße 50, Berlin 14197 Germany; ∥ Department of Obstetrics and Gynecology, 285569 Kenyatta National Hospital, Ngong Road, P.O. Box 20723, Nairobi 00202, Kenya; ⊥ Cancer Treatment Centre, Kenyatta National Hospital, Ngong Road, P.O. Box 20723, Nairobi 00202, Kenya; # Texas Cancer Center, Keri Road off Mbagathi Way, P.O. Box 13, Nairobi 00202, Kenya

**Keywords:** *N*-glycans, ovarian cancer, chemotherapy response, cancer biomarkers, benign ovarian disease, African ethnicity, permethylation, MALDI-TOF mass spectrometry

## Abstract

Epithelial ovarian cancer (EOC) remains the most lethal form of cancer despite improvements in surgical techniques and therapeutic interventions over recent decades. The high mortality rate is largely associated with a lack of sensitive and specific early diagnostic biomarkers to allow timely intervention. Hence, the identification and validation of novel noninvasive biomarkers for primary diagnosis and for disease monitoring is of high importance. Malignant transformations of *N*-glycosylation have been reported across various cancer types including EOC, but little is known about the *N*-glycome of African populations. In this work, we investigated aberrant *N*-glycosylation for the first time in an African EOC cohort comprising primary patients and those undergoing chemotherapy. In this pilot study, the *N*-glycome of African EOC and controls was comparable to those previously found in European cohorts. Of importance, high-mannose *N*-glycans increased with response to treatment in early chemotherapy cycles, and complex-type sialylated fucosylated *N*-glycans decreased, especially in the late chemotherapy cycles. Interestingly, the glycan-based index that we previously developed to detect primary EOC was more sensitive and specific than the routine diagnostic biomarker to identify primary EOC and to monitor chemoresponse in the early phase of the treatment.

## Graphical abstract

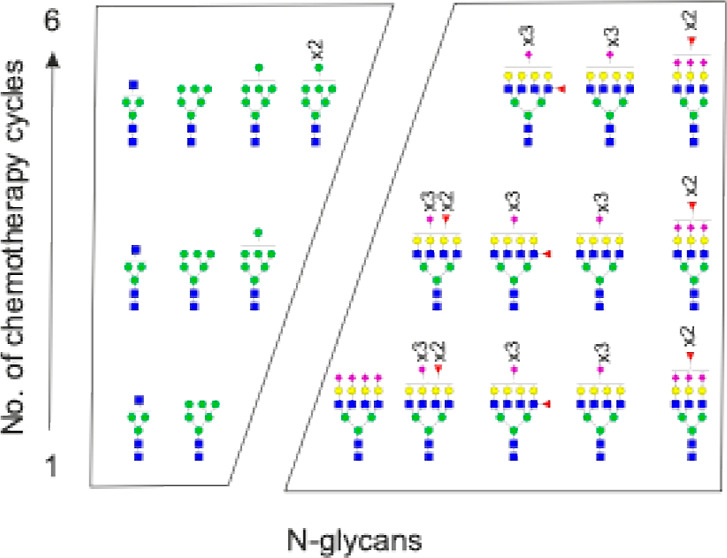

## Introduction

Although surgical techniques and therapies have improved over the past decades, ovarian cancer (OC) remains the most lethal malignancy among gynecological cancers.^1^ The high death rates due to OC arise from its propensity to misdiagnosis, as its symptoms are unspecific in the early stage (FIGO stage I and II) and lack accurate early diagnostic biomarkers. A majority of patients are diagnosed in late-stage (FIGO stages III and IV) of OC when cancer has already metastasized and treatment options have greatly diminished. Furthermore, resistance to chemotherapy and the presence of malignant ascites among epithelial OC patients have also been associated with poor prognosis and early death.^2,3^ In both developed and underdeveloped countries, more than 75% of OC patients are regrettably diagnosed in the third and fourth stages.^2,3^ Surgical debulking followed by chemotherapy remains the current standard front-line treatment for OC.^2,3^ A combination of platinum and paclitaxel is the standard first-line chemotherapy treatment approach usually administered in cycles of 3 weeks or carboplatin every 3 weeks and paclitaxel weekly, for six cycles. Unfortunately, patients may experience intrinsic or acquired resistance to chemotherapy. The most prominent mechanisms of platinum resistance include reduced drug uptake and increased efflux, enhanced DNA adduct repair, and increased drug inactivation by glutathione.^4,5^ Despite the availability of new treatment approaches, such as immunotherapy, resistance to chemotherapy continues to significantly impact the survival of OC patients. Indeed, despite their success in treating several tumor types including melanoma and lung cancer, the novel therapies arresting immune checkpoint pathways are not recommended for OC patients when used as monotherapies because the results of clinical studies have so far been disappointing.^5,6^ Cancer Antigen 125 (CA125), a heavily glycosylated mucin, is the routine biomarker used for OC detection, monitoring response to treatment and detecting relapse.^7,8^ CA125 is also elevated in some physiological states such as pregnancy and menses^9,10^ and pathological conditions such as liver diseases, endometriosis, and benign ovarian diseases (BOD),^9,11^ resulting in low sensitivity and specificity. Additionally, CA125 is not overexpressed in about 20% of the OCs^10^ and does not correlate with the prediction of platinum sensitivity or resistance.^7,8^

Glycosylation, the most frequent post-translational modification, does not only play a crucial role in the functionality and stability of proteins^12^ but is also modulated upon malignancy.^13–19^ *N*-Glycan changes previously reported for serum glycoproteins in EOC patients include decreases in the abundance of high-mannose and increases of tri- and tetraantennary sialylated and fucosylated *N*-glycans.^13,20,21^ Previously, our research group profiled permethylated *N*-glycans from serum samples of two European EOC cohorts by MALDI-TOF-MS and identified 11 altered *N*-glycans that were of statistical significance.^13,20^ As a result, we combined the significantly expressed glycan areas into a ratio named the “GLYCOV” score, which was more performant to differentiate EOC from BOD and healthy subjects than CA125 was.^13,20^ In addition, we also reported *N*-glycosylation changes in EOC patients on acute-phase proteins that included core-fucosylated biantennary *N*-glycans on α1-acid glycoprotein, increased antennarity and Lewis^X^ motif on haptoglobin, α1-antitrypsin, and α1-antichymotrypsin as compared with controls.^22^ Recently, we demonstrated an increase of α2,6-sialylation in OC tumor regions and elevated α2,3-sialylation in nontumor regions as well as tumor stroma.^21^ Similar findings of modulated *N*-glycosylation features in EOC patients have also been reported by other studies from different research groups.^15,23,24^ In addition, other glycosylation changes were published for other forms of cancer, as well. For instance, decreased expression of high-mannose and bigalactosylated biantennary *N-*glycans was measured for gastric cancer patients, while the nongalactosylated biantennary *N-*glycans were increased.^25^ Saldova et al. reported a significant increase of core-fucosylated biantennary and α2–3-linked sialylated *N*-glycans in prostate cancer patients compared to the benign prostate hyperplasia subjects.^26^ In pancreatic cancer, branching and antennary fucosylation were increased whereas high-mannosylation was not of statistical relevance.^18^ More importantly, *N*-glycosylation changes have been linked to major events in malignancy such as cancer growth, progression, and metastases.^14,27,28^ Kyselova et al. observed elevated sialylated and fucosylated glycans in breast cancer, which showed consistency with disease progression.^16^

Only a few studies have attempted to investigate the potential of *N*-glycome changes upon EOC malignancy as markers for monitoring the response to chemotherapy or addressing chemoresistance. Zahradnikova et al. identified six tissue *N*-glycans with characteristic bisecting, tetraantennary structures bearing sialic acid and/or fucose residues, which could potentially be used as markers of resistance to chemotherapy in OC patients.^29^ In another study, the serum of breast cancer patients changed in the abundance of high-mannose, core fucose, and galactose following chemotherapy administration, suggesting a response to chemotherapy.^17^ Recently, Zhao et al. observed different glycosylation patterns for Lewis-type biantennary, triantennary trisialylated, and Lewis-type triantennary glycans that differ between OC chemotherapy responders and nonresponders,^30^ but the glycome was not monitored during chemotherapy.

In the present work, we investigated the *N*-glycome from African EOC patients, for which samples were collected at the time of the first diagnosis. They were compared with age-matched BOD samples. In addition, serum from African EOC patients was collected at different cycles of chemotherapy as cross-sectional samples. The *N*-glycans released from these samples were compared with the *N*-glycans from primary African EOC subjects. To the best of our knowledge, this is the first time that serum glycome has been reported for a population of African ethnicity.

## Materials and Methods

All of the chemicals were purchased from Sigma-Aldrich (St. Louis, MO, USA) unless stated otherwise.

### Recruitment of Study Participants

We recruited an African cohort comprising 53 histologically confirmed EOC patients and 46 BOD patients (Supporting Information, Table S1). Recruitment took place in three Kenyan Hospitals within Nairobi city, namely, Kenyatta National Hospital (KNH), Texas Cancer Centre, and St. Mary’s Hospital Lanǵata, as per the ethical vote obtained from Kenyatta National Hospital/University of Nairobi ethics review committee (KNH/UON-ERC) reference no P701/12/2017. All the samples were collected from adult women of 18 years or more after giving their written informed consent. Of the 53 EOC patients, 19 were primary EOC patients (pretreatment group), while 34 were chemotherapy responders who had already undergone various sessions of chemotherapy (chemo) cycles that comprised carboplatin and paclitaxel (Supporting Information Figure S1A). Serum samples were collected into 5 mL vacutainers with a serum clot activator (Becton, Dickinson GmbH, Heidelberg, Germany). Samples were allowed to stand at room temperature between 30 and 120 min before centrifugation at 1,200*g* for 15 min. The separated serum aliquots were then stored in Eppendorf tubes at −80 °C until their shipment to Berlin, Germany. The necessary shipment approvals were obtained from the respective agencies in Kenya: KNH/UoN-ERC ref no., KNH-ERC/shipment/40, Ministry of Health Kenya, ref. No. MOH/F/HRD/01/VOL.11 and the Kenya Pharmacy and Poisons board export permit ref. no. CD2021000PPB321J0002550623. In Germany, the Ethical Commission of the Charité-Universitätsmedizin Berlin approved the analysis of the samples (approval number EA4/071/19).

### CA125 Analysis

Measurements of CA125 were done on a Cobas e 801 immunoassay system (Roche Diagnostics GmbH, Penzberg, Germany), a high-throughput fully automated immunochemistry module designed to carry out electrochemiluminescence sandwich immunoassays. The reagent used was an Elecsys CA125 II reagent (Roche diagnostics GmbH, Penzberg, Germany). The normal CA125 cutoff value was set at 35 kU/L for pre- and postmenopausal women.

### *N*-Glycan Release, Purification, and Permethylation

*N*-Glycans were released from serum samples and purified as described previously (Supporting Information Figure S1B).^13,20,31^ Briefly, 10 μL of serum was diluted in 2 μL of 200 mM phosphate buffer (pH 6.5). Serum glycoproteins were then reduced by adding 2.5 μL of 200 mM dithioerythritol (DTE) and incubated at 60 °C on a shaker for 45 min. Afterward, 10 μL of iodoacetamide was added and serum samples were left to alkylate for 1 h at room temperature in darkness. Subsequently, the reaction was stopped by the addition of the excess DTE. *N*-Glycans were then enzymatically released from glycoproteins using PNGase F (200 mU, *N*-Zyme Scientifics, Doylestown, PA, USA) for 16 h at 37 °C. The following day, *N*-glycans were isolated and desalted using C18 cartridges and porous graphitized carbon columns, respectively (both purchased from Alltech, Deerfield, IL, USA). The eluates were collected in Eppendorf tubes and then dried under a reduced atmosphere by centrifugal evaporation. *N*-Glycans were finally permethylated to neutralize sialic acids and to improve *N*-glycan ionization during MALDI-TOF measurements.^32^

### MALDI-TOF Measurements

Permethylated *N*-glycans were dissolved in 10 μL of 75% aqueous acetonitrile. Equal volumes (0.5 μL) of *N*-glycans and the super 2,5- dihydroxybenzoic acid matrix were spotted in triplicate on the ground steel target (Bruker Daltonics, Bremen, Germany). A glucose ladder was used for calibration, and measurements were made on an Ultraflex III (Bruker Daltonics, Bremen, Germany) in reflector positive ionization mode in the mass range of 1000–5000 Da. For each spectrum generated, at least 4000 laser shorts were recorded; baseline correction and peak picking were carried out using flexAnalysis (Bruker Daltonics, Bremen, Germany). *N-*Glycan spectra were analyzed using GlycoPeakfinder, and *N*-glycan cartoons were generated with GlycoWorkbench.^33,34^

### 2-AB-HPLC

*N*-Glycans were labeled with 2-aminobenzamide (2-AB), as described previously with small modifications.^31^ A 2AB-glucose ladder, which was prepared in parallel, was used as an external standard during HPLC measurements. In short, *N*-glycans, released from 5 μL serum, were labeled overnight at 37 °C using a solution containing 1 M 2AB and 0.24 M 2-picolinborane in acetic acid/methanol (1/9, v/v). The following day, samples were adjusted to 80% acetonitrile and applied to self-made cotton microcolumns, preconditioned with 3 × 40 μL of milli-Q water and then 3 × 40 μL of 80% ACN containing 0.1% TFA. After applying samples, microcolumns were washed 3 times with 40 μL of 80% ACN containing 0.1% TFA in order to remove the unreacted label. The derivatized *N*-glycans were then eluted with 6 × 40 μL of Milli-Q water. Samples were finally dried by centrifugal evaporation and reconstituted in 30 μL of water, from which 8 μL was taken for HPLC measurements. Separations were performed using an Ultimate 3000 (Dionex, Germany) equipped with a RS fluorescence detector (Dionex, Germany) with excitation and emission wavelengths of 330 and 420 nm, respectively. *N-*Glycan profiling was achieved at a flow rate of 0.75 mL/min with a XBridge Premier Glycan BEH Amide column (2.5 μm; 4.6 mm × 100 mm; Waters; USA) and a temperature of 20 °C. The linear gradient consisting of 100 mM ammonium formate (pH 4.5) (solvent A) and 100% ACN (solvent B) was set as follows: samples were injected in 22% A, and then the proportion of A was increased to 64% in 45 min. After maintaining 64% A for 10 min, the initial conditions were restored and maintained for 8 min. *N*-Glycans were assigned as previously^31^ using the GlycoBase database.^35^

### Statistical Analysis

Data analysis was performed using SPSS version 28 (SPSS Inc., Chicago, IL, USA). Mann–Whitney *U* test was used to compare the expression patterns of the detected *N*-glycans between the different patient groups, which were then presented as medians, range, and p-values. The *N*-glycan index GLYCOV previously established was calculated as follows: (sum of relative areas of *m*/*z* 3776.9, 3951.0, 4226.1, 4400.2, 4587.3, 4761.4, and 4935.5)/7*4/(sum of relative areas of *m*/*z* 1579.8, 1783.9, 1988.0, and 2192.3).^13^ Receiver Operating Curves (ROC) were built for the *N*-glycans that were of statistical significance. The corresponding values of the area under the curve (AUC, 95% C.I) were used to describe the accuracy levels of assessing EOC diagnosis and of monitoring response to chemotherapy. AUC values of ROC curve >0.9 indicated a “high accuracy” outcome, values of 0.8 to 0.9 meant “good accuracy”, 0.7 to 0.8 indicated moderate accuracy, while values 0.5 and 0.7 were interpreted as “uninformative”. Box plots were generated to describe the distribution of the *N*-glycan index and CA125 values in the patients’ three clusters of chemotherapy intake.

## Results

We present the first evaluation of the *N*-glycome profile in an African EOC cohort, testing its clinical utility in monitoring the chemotherapy response. We also sought to validate our previous findings of early EOC diagnostic *N*-glycan signatures from Caucasian cohorts in African patients.^13,20^

### Method Repeatability Test

Intra- and interday reproducibility were verified by analyzing the same serum sample in triplicate on a single day and on three consecutive days (Supporting Information Figure S2). Four low-abundance *N*-glycan signals corresponding to high-mannose *N*-glycans (*m*/*z* 1579.8, 1783.9, 1988.0, and 2192.1) were selected for this evaluation because they had previously been described by our research group as biomarker signatures of EOC among Caucasians. The mean coefficients of variation are below 10%, indicating the good reproducibility of our analytical workflow.

### *N*-Glycan Diagnostic Signatures of Primary EOC from African Ethnicity

We cleaved, isolated, and analyzed *N*-glycans from an African cohort consisting of 53 EOC patients and 46 BOD subjects by MALDI-TOF-MS. A total of 46 signals were detected and assigned to permethylated *N*-glycans (Supporting Information Table S2). Figure 1 shows a representative MALDI-TOF spectrum of *N*-glycans from a control subject against an EOC patient of African ethnicity. A nonparametric Mann–Whitney *U* test was applied to the relative intensities of the *N*-glycan peaks to describe the differences in their expression between primary EOC patients and BOD subjects.

**Figure 1:**
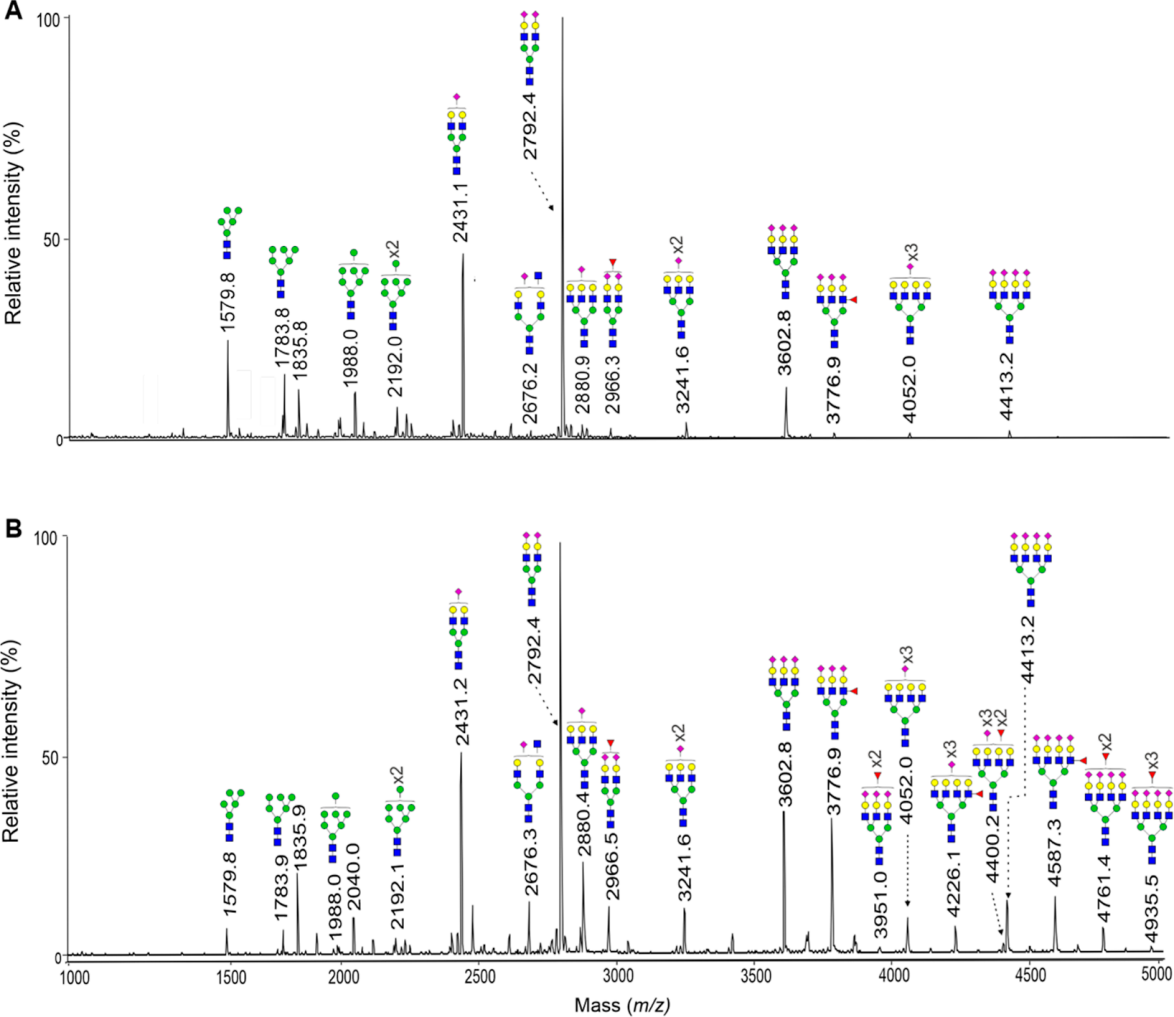
Representative MALDI-TOF mass spectra of the permethylated *N*-glycans from (A) a BOD subject and (B) a primary EOC patient. Measurements were carried out in positive-ion mode and molecular ions are present in their [M + Na]^+^ form. The monosaccharides are depicted as follows: Man, green circle; Gal, yellow circle; GlcNAc, blue square; Fuc, pink triangle; Neu5Ac, pink diamond.

A total of 39 *N*-glycans were differentially expressed in primary EOC patients as compared to their control counterparts (*p* < 0.05) (Supporting Information Table S2). In the mass range *m*/*z* < 3600, 27 signals, corresponding mainly to high-mannose, monoantennary, and biantennary *N*-glycans, had decreased intensities in EOC patients. On the opposite, in the mass range *m*/*z* > 3600, 12 signals, assigned to complex-type tri- or tetraantennary *N*-glycans being sialylated and fucosylated, had increased intensities in EOC patients as compared to control subjects (Supporting Information Table S2). Interestingly, the 11 *N*-glycans constituting the glycan index GLYCOV^13,20^ that was previously developed by our research group using Caucasian cohorts are part of the statistically significant *N*-glycans in the present African cohort. The 11 *N*-glycans constituting the glycan index GLYCOV included four high-mannose *N*-glycans (*m*/*z* 1579.8, 1783.9, 1988.0, and 2192.1), which were decreased in primary EOC, and seven increased complex-type *N*-glycans (*m*/*z* 3776.9, 3951.0, 4226.1, 4400.2, 4587.3, 4761.4, and 4935.5). We set a cutoff value at <1.82 to denote a normal *N*-glycan index value, as obtained from the coordinates of the ROC curve. From the foregoing, the *N*-glycan index was highly accurate in discriminating EOC patients from the control subjects (AUC 0.94, [C.I 0.880–1.00], SP, 98%) compared to CA125 (AUC 0.88, [C.I 0.801–0.961, SP, 67%]) (Supporting Information Figure S3). No additional discriminatory advantage was observed when the *N*-glycan index was used in combination with CA125 (AUC 0.94, C.I [0.880–1.000]).

### *N*-Glycans’ Signatures for Monitoring Chemotherapy Response in EOC

The total serum *N*-glycome of EOC patients undergoing chemotherapy was then analyzed to determine its potential utility in monitoring patients’ response to chemotherapy. To this end, EOC patients were subdivided into three categories based on the number of chemotherapy cycles received at enrollment into the study: pretreatment, 1–3 chemo cycles, and 4–6 chemo cycles. Figure 2 shows a representative MALDI-TOF mass spectrum of the *N*-glycans from each patient category.

**Figure 2:**
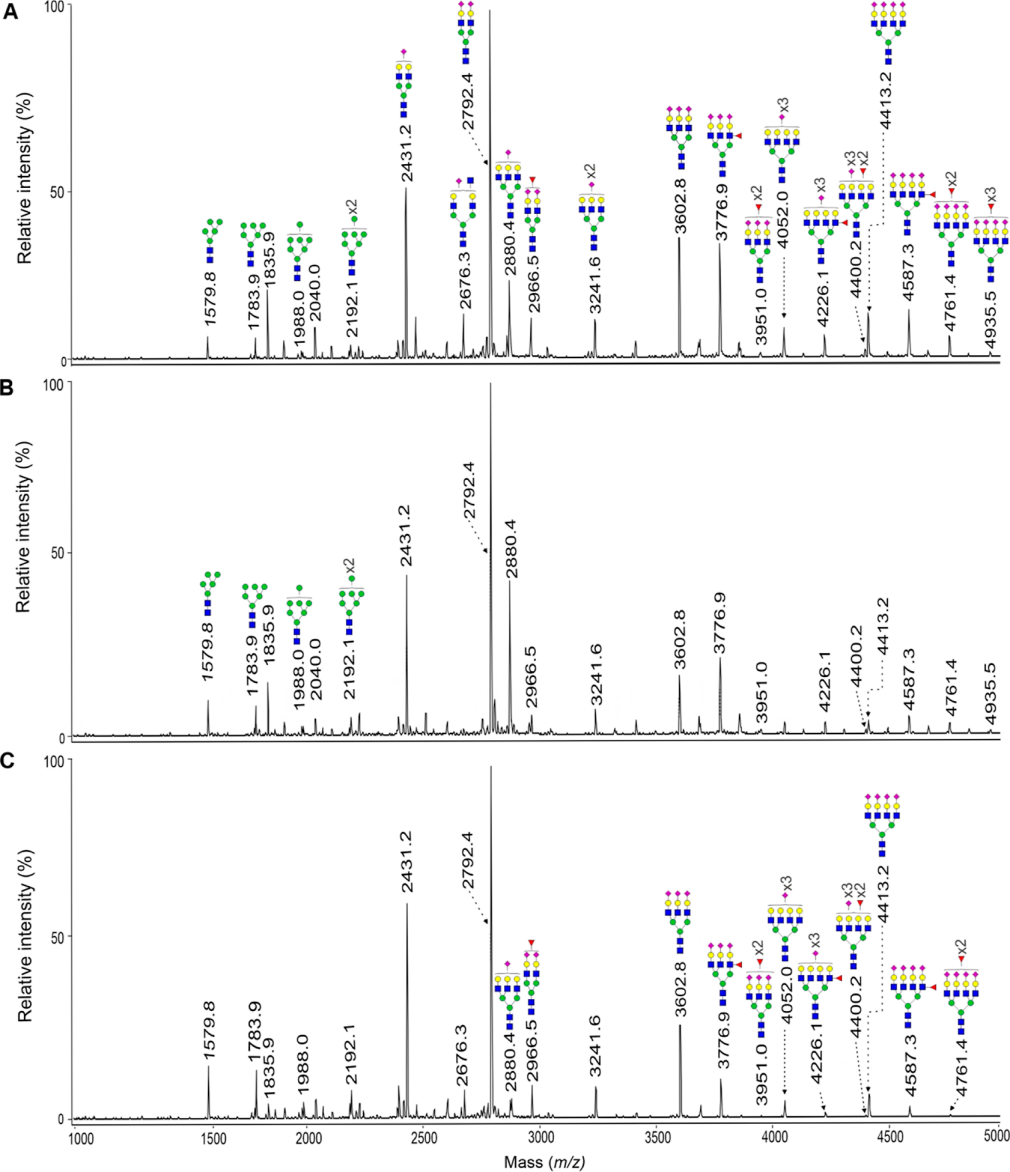
Representative MALDI-TOF mass spectra of the permethylated *N*-glycans from EOC patients: (A) pretreatment; (B) 1–3 chemo cycles; and (C) 4–6 chemo cycles. The *N*-glycans of *m*/*z* < 3600, which include the high-mannose-type, were increased after 1–3 and 4–6 chemo cycles (B,C) as compared to the pretreatment group (A). Conversely, the *N*-glycans of *m*/*z* > 3600, which include complex-type fucosylated and sialylated *N*-glycans, were decreased with the increasing number of chemo cycles (B,C). Measurements were performed in positive ionization mode and molecular ions are present in their [M + Na]^+^ form. Man, green circle; Gal, yellow circle; GlcNAc, blue square; Fuc, pink triangle; Neu5Ac, pink diamond.

Sample-independent Kruskal–Wallis test was used to compare medians across the groups, and ROC curves were built for each of the 27 statistically significant *N*-glycans to determine the accuracy of differentiating the patients in the three categories. Seventeen *N*-glycans (of *m*/*z* < 3600) showed a statistically significant increase (*p* < 0.05) and AUC> 0.7 as the number of chemo cycles increased (Supporting Information Table S3). They comprised the *N*-glycans of *m*/*z* 1579.8, 1783.9, 1988.0, 1620.8, 1416.7, 1982.0, 2390.2, 2285.2, 2315.2, 2489.3, 2850.4, 1661.8, 2070.0, 2519.3, 2693.4, 2880.4, and 3054.5, boldly highlighted by their respective AUCs. The 17 *N*-glycans signatures (of *m*/*z* < 3600) demonstrated response to chemotherapy agents within the first three cycles of chemotherapy when compared to the primary EOC baseline group. The patients that had taken 4–6 chemo cycles had the highest peak increases, affirming a positive response of patients to chemotherapy. On the other hand, nine complex-type *N*-glycans (of *m*/*z* > 3600) whose intensities were elevated in pretreatment EOC patients, were significantly decreased after 1–3 and 4–6 chemo cycles (*p*-value <0.05 and an AUC value >0.70). They comprised the *N*-glycans of *m*/*z* 3415.7, 3776.9, 3864.9, 3951.0, 4226.1, 4400.2, 4587.3, 4761.4, and 4935.5 (Supporting Information Table S4). It should be noted that these nine *N*-glycans could not differentiate between primary EOC patients and EOC patients who received 1–3 chemo cycles. MALDI-TOF data were verified by 2AB-HPLC. To this end, released *N*-glycans were labeled with 2-AB and then measured by HPLC equipped with fluorescence detection (Supporting Information Figure S4). An increase of high-mannose *N*-glycans and a decrease of sialylated *N*-glycans were observed as the number of chemo cycles increased.

As the 11 *N*-glycans that are part of the glycan index GLYCOV previously established by our research group for EOC in Caucasian cohorts^13,20^ were of statistical relevance, GLYCOV was computed and evaluated for its performance in distinguishing patients across the three chemotherapy categories. The log2-transformed values of the *N*-glycan index and CA125 for each patient category were plotted in a box plot, and the differences between them were statistically tested (Figure 3). The *N*-glycan index showed statistically significant decreases between the three patient categories, the pretreatment group having the highest values and 4–6 chemo cycles having the lowest values (Figure 3A). On the other hand, CA125 could differentiate pretreatment from 4–6 chemo cycles but not pretreatment from 1 to 3 chemo cycles (Figure 3B).

**Figure 3:**
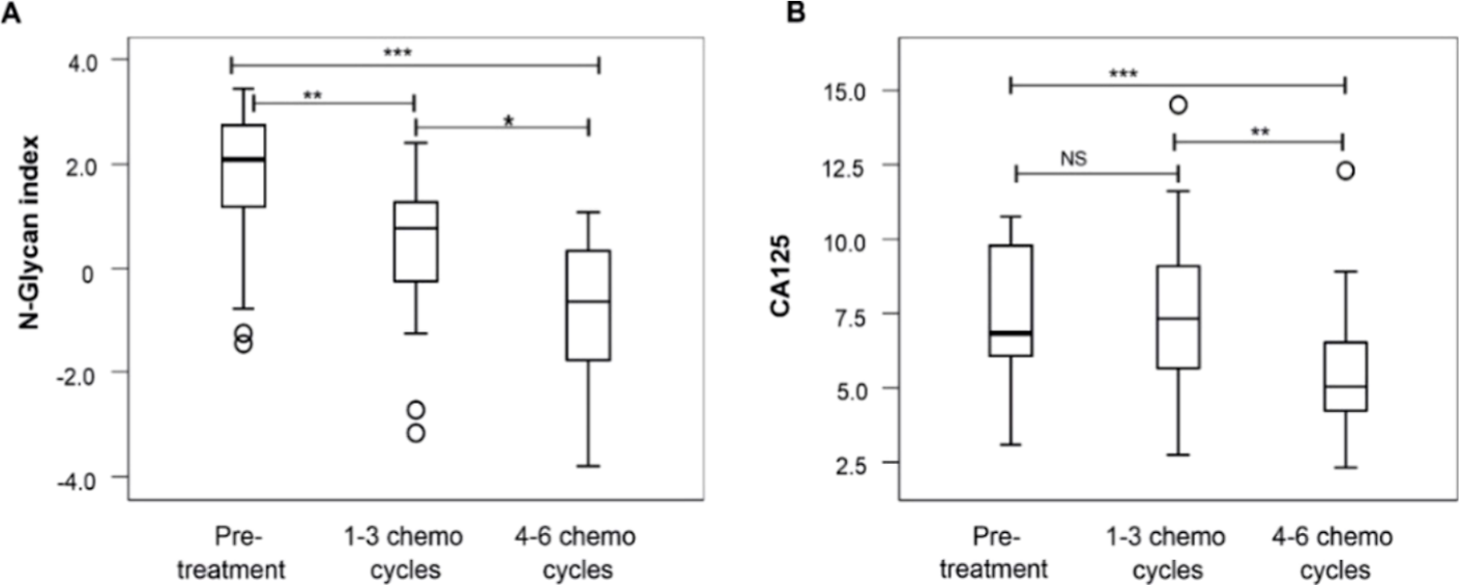
Box plots of log2-transformed values of (A) GLYCOV, the *N*-glycan index and (B) CA125 analyzed by the Mann–Whitney *U* test (*p < 0.05, **p < 0.01, and ***p < 0.001).

Next, we built ROC curves for both cancer biomarkers; the *N*-glycan index demonstrated improved accuracy in distinguishing EOC patients across the three treatment categories compared to CA125 (Figure 4). Indeed, AUC values were 0.77 for the *N*-glycan index versus 0.54 for CA125 when pretreatment patients were compared to patients undergoing 1–3 chemo cycles (Figure 4A). When pretreatment was compared with 4–6 chemo cycles, AUC of the *N*-glycan index was as high as 0.89 versus 0.8 for CA125 (Figure 4B).

**Figure 4:**
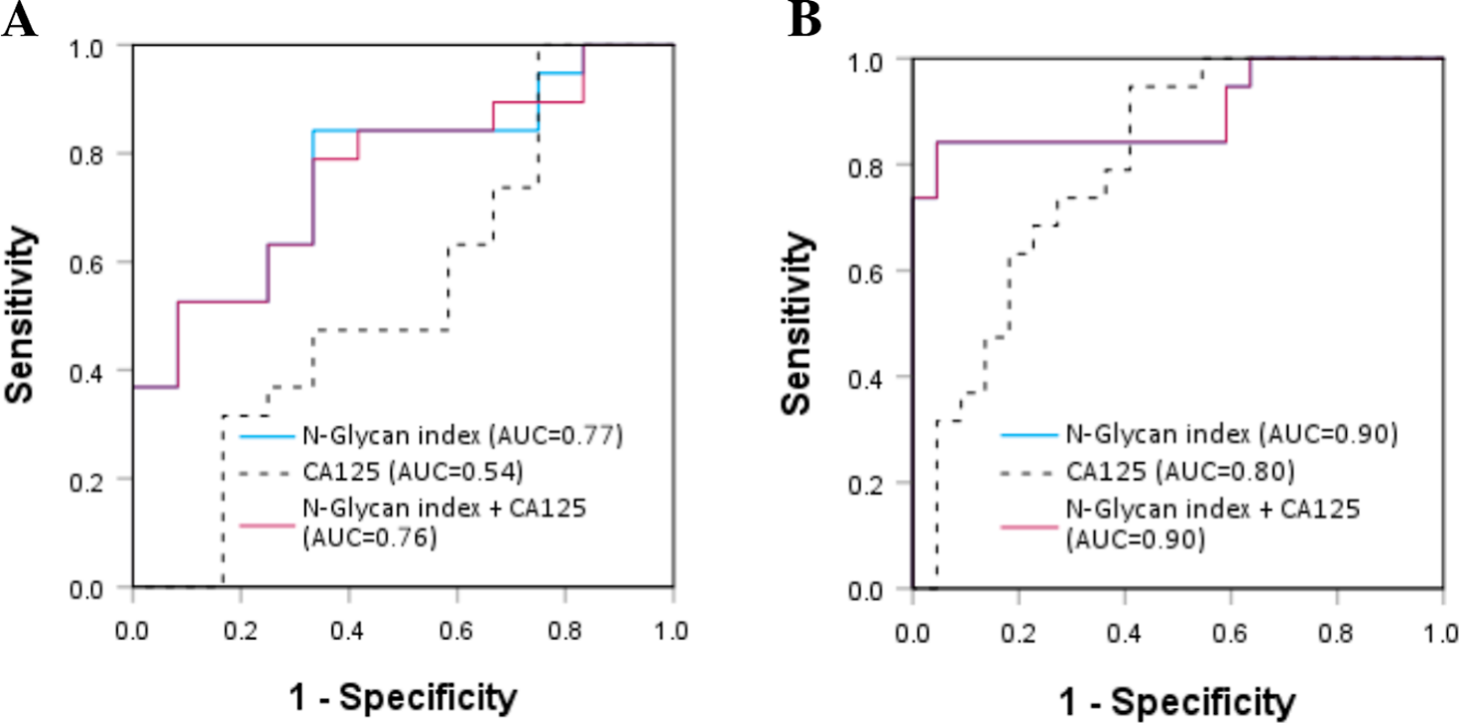
ROC curves comparing CA125 and the glycan index to assess response to chemotherapy: (A) pretreatment versus 1–3 chemo cycles, and (B) pretreatment versus 4–6 chemo cycles.

## Discussion

In the past years, various independent research groups including our laboratory have studied extensively EOC-related changes of the *N*-glycome,^13,15,20,24,36–39^ but mostly using Caucasian and Asian populations. The data on neoplastic glycosylation changes of EOC in African populations had not been documented so far, in contrast to the Caucasian and Asian ethnicities. Therefore, for the first time, we provided in this work data on the EOC-mediated alterations of the total *N*-glycome in an African population to identify signature biomarkers for primary diagnosis of EOC as well as for monitoring patients’ response to chemotherapy. The subjects recruited into this study were largely stemming from urban and peri-urban settings and hence nearly of relatively similar social and economic demographics to that of the Caucasian cohorts used for the comparison analysis. The similarities in the variables of the two comparative groups reinforce the resultant findings thereof.

Interestingly, African BOD subjects had *N*-glycan profiles that were very comparable with our previously reported European control profiles.^13,20^ Surprisingly, although Kenya borders Ethiopia, control subjects from both countries seem to have different *N*-glycosylation patterns.^40^ Gebrehiwot and co-workers reported increased intensities for high-mannose, core-fucosylated, multiantennary, and multisialylated glycans in Ethiopian subjects when compared with US, Indian, and Japanese healthy subjects, which we did not observe in this work.^40^ It should be noted that the method used in that article prior and posterior to *N*-glycan release was different from ours, preventing a direct comparison of the data. It is also worth noting that the total serum glycome, unlike the IgG glycome, is not significantly influenced by age.^41^ Pongracz and co-workers recently showed that the *N*-glycans that are of relevance in this present research work, namely, sialylated tri- and tetraantennary and high-mannose *N*-glycans, are not markedly associated with age.^42^ In addition, the *N*-glycome is temporally stable in single individuals.^43^

### *N*-Glycome Diagnostic Signatures for Primary EOC

The current analysis showed that *N*-glycome modulations resulting from the malignant EOC growth in individuals of African ethnicity were comparable to the ones previously found in European cohorts.^13,20,31,36,44–46^ We identified 39 *N*-glycan biomarker signatures that successfully discriminated primary EOC patients from BOD subjects. The reported *N*-glycosylation changes include a decrease of high-mannosylation and an increase of antennarity, sialylation, and fucosylation. Using nano-HPLC-chip-TOF-MS, Hua and colleagues previously identified 26 differentially expressed *N*-glycans between the EOC patients and the healthy controls, highlighting decreased abundances of high-mannose and hybrid *N*-glycans in EOC while also reporting increases in peaks of complex-type *N*-glycans.^24^ Modulated expression of high-mannose is a key feature of many cancers, as corroborated by many studies including the present one.^13,15,16,20,36,47,48^ In addition, elevated levels of free mannose were recently measured in the serum of EOC patients;^49^ however, the exact role played by high-mannosylation in tumor etiology is yet to be overtly elucidated. Alley et al. also reported increased abundances of tri- and tetraantennary complex-type *N*-glycans among EOC patients, while bisecting *N*-glycans were reduced.^37^ Furthermore, increased sialylation was also observed in the serum glycome from a mouse model of ovarian cancer.^44^ A decrease in high-mannose structures was similarly observed in gastric cancer patients,^25^ while fucosylated, sialylated, and branched *N*-glycans structures were increased in prostate, lung, pancreatic, and colorectal cancer.^18,50–52^ On the contrary to gastric and breast cancer,^16,25^ an increase of agalactosylated biantennary *N*-glycans is not observed in ovarian cancer, demonstrating the cancer specificity of glycome changes. Indeed, these glycan features have also been associated with the progression, tumor evasion, and metastasis of cancers including OC and with the overexpression of the corresponding glycosyltransferases, namely, *N*-acetylglucosaminyltransferase IV and V, the sialyltransferases ST3Gal I and ST6Gal I, and alpha-1,3-fucosyltransferase type VII.^46,53^ Moreover, glycosylation may also be influenced by nutrition, as already shown *in vitro*: adipose conditioning medium was previously found to increase α2,6- and α 2,3-linked sialylation of ovarian cancer cells.^54^ Sialic acid and fucose residues cap galactose residues, preventing the clearance of glycoproteins with the asialoglycoprotein receptor and allowing a longer circulation of acute-phase proteins in the bloodstream.^55–57^

The findings on the use of our *N*-glycan score, consisting of 11 *N*-glycan signatures, for primary diagnosing EOC in an African cohort, were concordant with our former analysis on Caucasian cohorts.^13,20^ Compared with CA125, the *N*-glycan index demonstrated excellent diagnostic accuracy and specificity. The specificity improved even further to 98% when the *N*-glycan index was used in conjunction with CA125. Glycosylation has previously been found to improve existing routine biomarkers, such as prostate-specific antigen (PSA) and CA125. Indeed, increased PSA fucosylation, as measured by binding to the lectin Aleuria Aurantia Lectin, is able to refine the diagnosis of prostate cancer.^58^ In addition, Llop and co-workers used PSA-sialylation to discriminate aggressive from nonaggressive prostate cancer.^59^ CA125 glycoforms containing the Sialyl-Thomsen-Friedenreich antigen were also used to predict the progression-free survival and relapse of high-grade serous EOC patients.^60^

### Utility in Chemotherapy Response Monitoring

The current analysis identified potential 27 *N*-glycans for monitoring chemoresponse in patients that had undergone 1–3 and 4–6 chemo cycles. The 27 *N*-glycans were originally differentially expressed in primary EOC, and they included the 11 *N*-glycans that constitute our GLYCOV score. Of importance to note is that the 27 *N*-glycans showed the efficacy of chemotherapy, whereby the high-mannose *N*-glycans were statistically increased within the period of administration of 1–3 chemo cycles whereas the complex-type, sialylated, and fucosylated *N*-glycans only statistically decreased from the fourth to sixth chemo cycles. Interestingly, our glycan index was able to differentiate the pretreatment group from the group of 1–3 chemo cycles, whereas CA125 was not able to do so. Both biomarkers were able to discriminate between the pretreament group and the group of 4–6 chemo cycles. As we used cross-sectional samples from responder patients to address treatment monitoring in this work, it is likely that false-negative samples are underestimated. Future studies should include a larger number of patients and treatment monitoring of the same patients over time to confirm the data. Although data on glycomic-based biomarkers for monitoring chemotherapy response in malignant patients is limited, our data is in line previous research.^30,61^ Evidence from a previous study by Miyahara et al. identified the high-mannose HexNAc_2_Hex_9_ as a potential biomarker for the efficacy of gemcitabine monotherapy treatment in unresectable advanced pancreatic cancer patients as well as for predicting patient survival.^61^ In another study, Zhao et al. collected and analyzed serum from EOC patients to predict resistance to chemotherapy.^30^ They identified the Lewis type HexNAc_4_Hex_5_dHex_1_Neu5Ac_2_, HexNAc_5_Hex_6_dHex_1_Neu5Ac_3_ and the trisialylated HexNAc_5_Hex_6_dHex_1_Neu5Ac_3_ as predictive markers for chemoresistance, α2–3 sialylation being increased whereas α2–6 sialylation was decreased.^30^ At the tissue level, Zahradnikova et al. had associated eight *N*-glycans with resistance to chemotherapy; they were complex-type mono- and biantennary *N*-glycans.^29^ Besides, similarly to the current analysis, Saldova et al. also found an increase of the otherwise decreased high-mannose HexNAc_2_Man_6_ in breast cancer patients upon initiation of chemotherapy.^17^

## Conclusions

Our glycan-based biomarker was more efficient than CA125 to diagnose primary EOC and to monitor the response to therapy in an African cohort. While larger multicenter studies are needed to confirm the present data without concern for ethnic variations in glycosylation, the current findings justify the use of a common *N*-glycan biomarker approach for diagnosing EOC in a mixed ethnic population of African and Caucasian patients.

## Supplementary Material



## Data Availability

The raw data is available on GlycoPost with accession number: GPST000583.^62^

## Notes

Overview of the serum samples and analytical workflow used in this article, intra- and interday reproducibility, ROC curves of the *N*-glycan index and CA125 biomarkers, representative 2AB-HPLC *N*-glycan profiles, demographics and clinical factors of the participants, differential *N*-glycan expression of primary EOC versus BOD subjects, *N*-Glycans (*m*/*z* < 3600), decreased in primary EOC patients, increased upon intake of chemotherapy, and *N*-glycans (*m*/*z* > 3600), increased in primary EOC patients, decreased upon intake of chemotherapy (PDF)
