# Supplementary material for: High-Mannose N‑Glycans To Monitor Early Response to Chemotherapy in African Epithelial Ovarian Cancer Patients—A Pilot Study
Source: J Proteome Res. 2025 Dec 10;25(1):99–108. doi: 10.1021/acs.jproteome.5c00442 (PMC12772126; doi:10.1021/acs.jproteome.5c00442)
Supplement: Supplementary file 1 [file pr5c00442_si_001.pdf]

Supporting information

High-mannose N-glycans to monitor early response to chemotherapy in African epithelial ovarian cancer patients – a pilot study

Francis M. Wanyama[1,2,], Obinna Umeh[1], Karina Biskup[3], Rudolf Tauber[1], Alfred Mokomba[4], Catherine Nyongesa[5,6] and Véronique Blanchard[1,3,*]*

[1] Institute of Diagnostic Laboratory Medicine, Clinical Chemistry and Pathobiochemistry, Charité – Universitätsmedizin Berlin, corporate member of Freie Universität Berlin, Humboldt-Universität zu Berlin and Berlin Institute of Health. Post: Augustenburger Platz 1, D-13353 Berlin, Germany obinna.umeh@charite.de, rudolf.tauber@charite.de, veronique.blanchard@charite.de

[2] Department of Human Pathology, Thematic Unit of Clinical Chemistry, University of Nairobi, P.O. Box 19676, 00202 Nairobi, Kenya, francis.wanyama@uonbi.ac.ke

[3] Department of Human Medicine, Medical School Berlin, Rüdesheimer Straße 50, 14197 Berlin karina.biskup@medicalschool-berlin.de, veronique.blanchard@medicalschool-berlin.de

[4] Department of Obstetrics and Gynecology, Kenyatta National Hospital, Ngong road, P.O. Box 20723, 00202 Nairobi, Kenya, mokombadoc@gmail.com

[5] Cancer Treatment Centre, Kenyatta National Hospital, Ngong road, P.O. Box 20723, 00202 Nairobi, cnyongesa@knh.or.ke

[6] Texas Cancer Center, Keri road off Mbagathi Way, P.O. Box 13, 00202 Nairobi, Kenya

Table of content

Page 2 **Figure S1.** Overview of the serum samples and analytical workflow used in this article.
Page 3 **Figure S2.** Intra- and inter-day reproducibility
Page 4 **Figure S3.** ROC curves of the *N*-glycan index and CA125 biomarkers.
Page 5 **Figure S4**. Representative 2AB-HPLC *N*-glycan profiles.
Page 6 **Table S1**. Demographics and clinical factors of the participants.
Page 7 **Table S2.** Differential *N*-glycan expression of primary EOC versus BOD subjects.
Page 10 **Table S3.** *N*-Glycans (m/z < 3600), decreased in primary EOC patients, increased upon intake of chemotherapy.
Page 13 **Table S4.** *N*-Glycans (m/z > 3600), increased in primary EOC patients, decreased upon intake of chemotherapy.

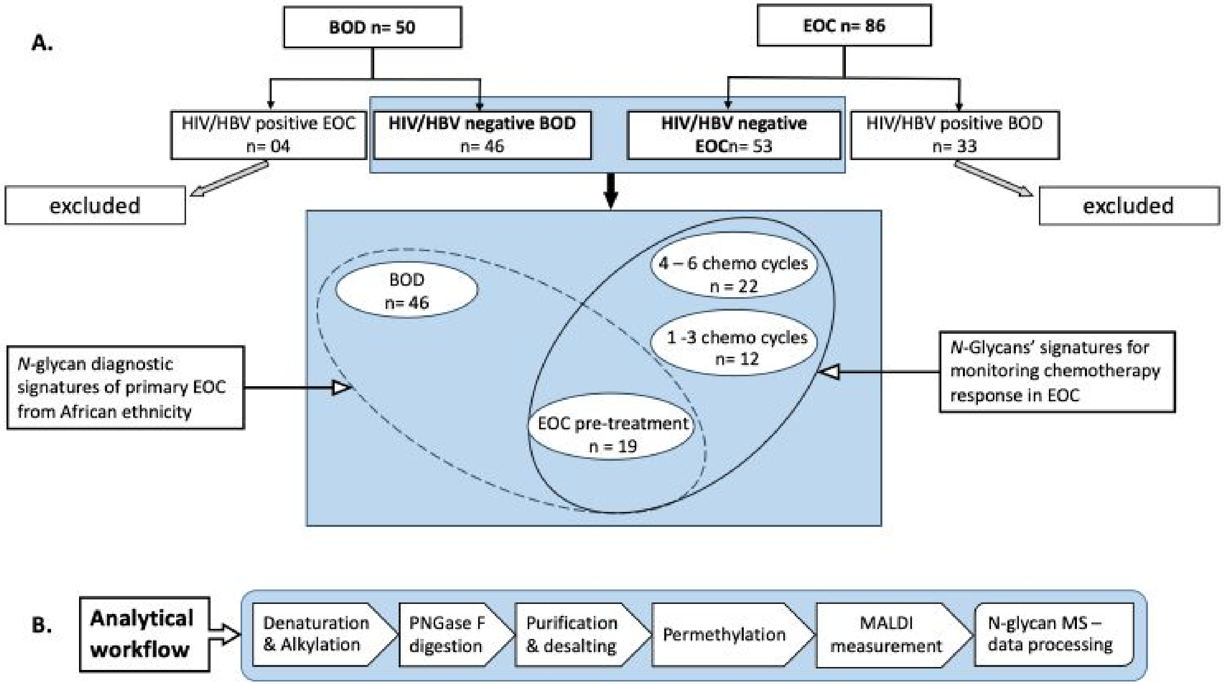

Figure S1. (A) Overview of the serum samples recruited in this study. (B) Analytical workflow used in this article.

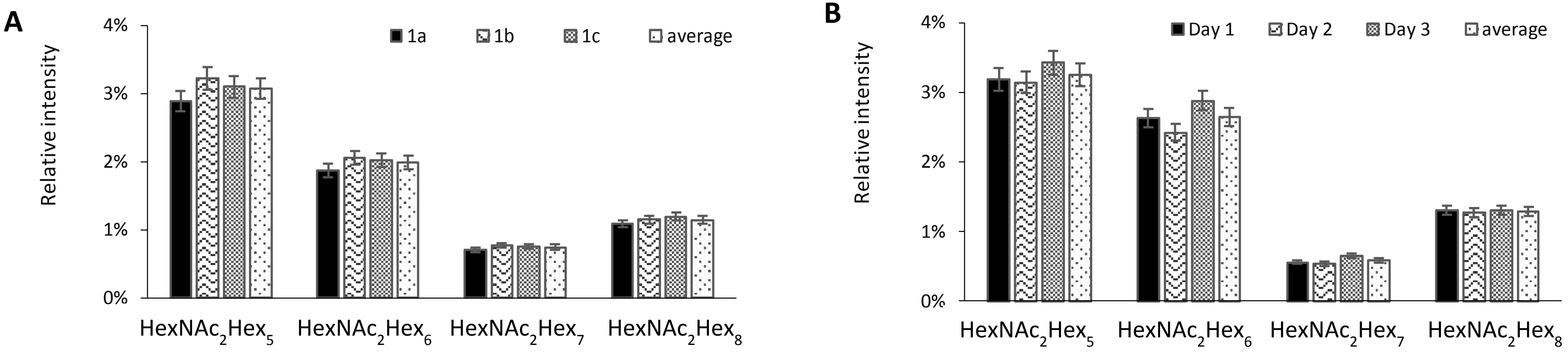

Figure S2. Intra- (A) and inter-day (B) reproducibility of the workflow used to prepare *N*-glycans. The relative intensities of the four high-mannose *N*-glycans previously described by our research group as potential signature biomarkers for EOC [1,2] were used in this experiment. Experiments were performed with one sample of primary EOC patient that was analyzed in triplicate within the same day (A) or on three consecutive days (B).

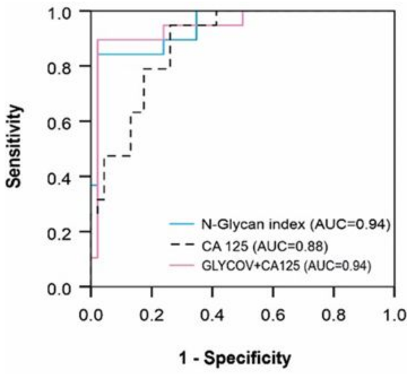

Figure S3. ROC curves of the *N*-glycan index and CA125 biomarkers showing their performance in discriminating EOC patients from BOD subjects.

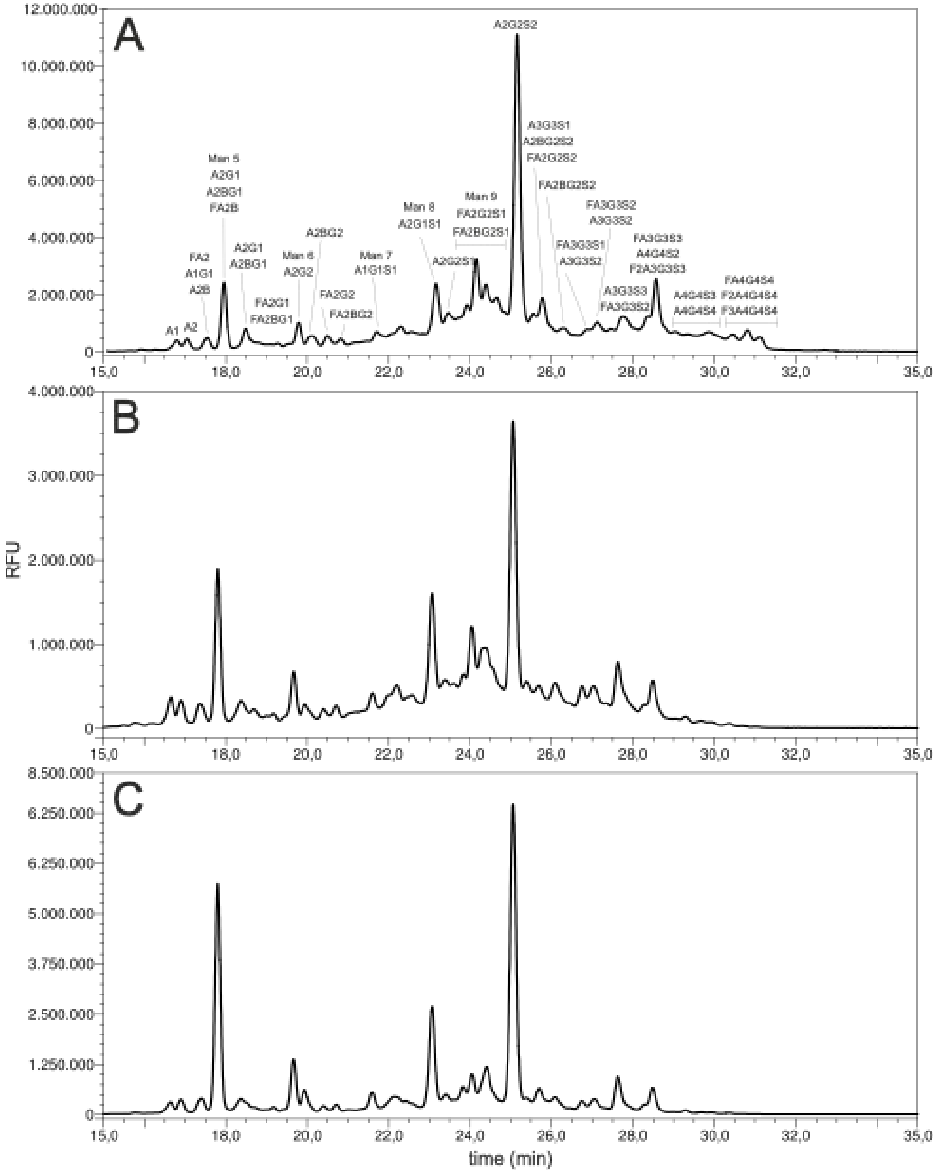

Figure S4. Representative 2AB-HPLC *N*-glycan profile from equivalent amounts of patient serum (A, pre-treatment; B, 1-3 chemotherapy cycles; C, 3-6 chemotherapy cycles). All *N*-glycans have a core consisting of two core GlcNAcs and three mannose residues. Manx, high-mannose where x is the number of mannose residues; F = core fucose; Ax, number of antennae; B, bisecting GlcNAc; Gx, number of galactose residues; Sx, number of sialic acids.

Table S1. Demographics and clinical factors of the participants. Chemotherapy is stratified into 3 categories depending on the number of chemotherapy cycles taken.

Characteristics	African cohort	
	**EOC (n = 53)**	**Control (n =46)**
Age (years)		
Range	20 – 81	18 – 74
Mean ±SD	50.6 ±16.5	36.5 ±11.6
Median	50	34
Inter quartile range	38 - 65	28 – 42.3
Tumor stage % (n)		
FIGO I & II (early stage)	17.4 (15)	
FIGO III & IV (late stage)	82.6 (38)	
Chemotherapy % (n)		
Primary (pre-treatment)	35.8 (19)	
On chemotherapy (total)	64.2 (34)	
1 – 3 chemo cycles	22.6 (12)	
4 – 6 chemo cycles	41.5 (22)	
CA125 (kU/L)		
Range	05 - 31214	4.2 - 998
Mean ±SD	991.6 ±4318.4	76.0 ±165.2
Median	92.4	25.9
Inter quartile range	77.2 – 1017	12.1 – 65.5

Table S2. Differential *N*-glycan expression of primary EOC versus BOD subjects. The 11 *N*-glycans that constitute the GLYCOV score are highlighted in bold. Medians were calculated from the relative intensities of each N-glycan in each cluster of study subjects: Min-Max is the minimum and maximum of relative intensities of the N-glycan peaks in each cohort: RI is the relative intensity of the N-glycan peaks. Bonferroni corrected p-values were computed to evaluate the difference in N-glycan expression between EOC patients and BOD subjects; $p \leq 0.005$ (0.05/11), $p \leq$ 9.1E-4 (0.01/11) and $p \leq$ 9.1E-5 (0.001/11 were considered to be statistically significant. AUC assessed the discriminatory accuracy of each N-glycan between EOC patients and BOD subjects.

			**Putative**	**BOD (n=47)**		**EOC (n=19)**		**p- value**	**AUC**
	**Formula**	***m/z***	**structure**	**Median**	Min-max	**Median**	Min-max		**(95% C.I)**
1	$HexNAc_3Hex_3$	1416.7	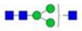	0.002	0.001-0.005	0.001	0.0004-0.003	<0.001	
2	**$HexNAc_2Hex_5$**	1579.8	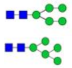	0.031	0.008-0.059	0.013	0.003-0.020	<0.001	0.94 (0.879 - 0.993)
3	$HexNAc_3Hex_4$	1620.8	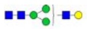	0.003	0.001-0.007	0.001	0.0003-0.002	<0.001	
4	$HexNAc_4Hex_3$	1661.8	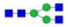	0.003	0.001-0.010	0.001	0.0003-0.004	<0.001	
5	**$HexNAc_2Hex_6$**	1783.9	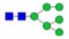	0.028	0.007-0.065	0.013	0.002-0.020	<0.001	0.91 (0.832 - 0.976)
6	$HexNAc_4Hex_3dHex_1$	1835.9	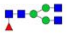	0.019	0.006-0.087	0.022	0.008-0.070	0.604	
7	$HexNAc_3Hex_4Neu5Ac_1$	1982.0	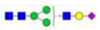	0.008	0.004-0.015	0.004	0.001-0.008	<0.001	
8	**$HexNAc_2Hex_7$**	1988.0	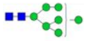	0.010	0.003-0.021	0.004	0.001-0.009	<0.001	0.92 (0.844 - 0.985)
9	$HexNAc_4Hex_4dHex_1$	2040.0	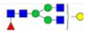	0.018	0.006-0.052	0.010	0.002-0.024	<0.001	
10	$HexNAc_4Hex_5$	2070.0	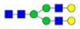	0.009	0.003-0.023	0.003	0.001-0.007	<0.001	
11	$HexNAc_5Hex_4$	2111.1	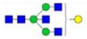	0.004	0.002-0.009	0.002	0.001-0.007	0.003	

12	**HexNAc$_2$Hex$_8$**	2192.1	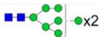	0.018	0.006-0.037	0.008	0.001-0.019	<0.001	0.91 (0.828 - 0.980)
13	HexNAc$_4$Hex$_4$Neu5Ac$_1$	2227.1	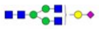	0.012	0.008-0.022	0.007	0.002-0.015	<0.001	
14	HexNAc$_4$Hex$_5$dHex$_1$	2244.1	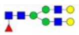	0.007	0.002-0.017	0.002	0.0003-0.006	<0.001	
15	HexNAc$_5$Hex$_4$dHex$_1$	2285.2	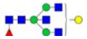	0.002	0.001-0.003	0.001	0.0002-0.002	<0.001	
16	HexNAc$_5$Hex$_5$	2315.2	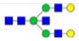	0.002	0.001-0.004	0.001	0.0002-0.002	<0.001	
17	HexNAc$_3$Hex$_6$Neu5Ac$_1$	2390.2	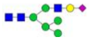	0.005	0.002-0.012	0.003	0.001-0.007	<0.001	
18	HexNAc$_4$Hex$_5$Neu5Ac$_1$	2431.2	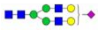	0.144	0.085-0.189	0.090	0.053-0.152	<0.001	
19	HexNAc$_5$Hex$_5$dHex$_1$	2489.3	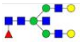	0.003	0.001-0.005	0.001	0.0002-0.003	<0.001	
20	HexNAc$_5$Hex$_6$	2519.3	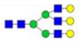	0.002	0.001-0.004	0.001	0.0002-0.002	<0.001	
21	HexNAc$_4$Hex$_5$Neu5Ac$_1$dHex$_1$	2605.3	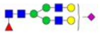	0.010	0.005-0.021	0.006	0.001-0.014	<0.001	
22	HexNAc$_5$Hex$_5$dHex$_2$	2663.3	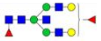	0.004	0.002-0.011	0.004	0.001-0.008	0.462	
23	HexNAc$_5$Hex$_5$Neu5Ac$_1$	2676.3	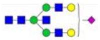	0.007	0.004-0.016	0.003	0.001-0.010	<0.001	
24	HexNAc$_5$Hex$_6$dHex$_1$	2693.4	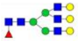	0.002	0.001-0.002	0.001	0.0002-0.002	<0.001	
25	HexNAc$_6$Hex$_6$	2764.4	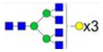	0.005	0.002-0.010	0.003	0.001-0.006	<0.001	
25	HexNAc$_4$Hex$_5$Neu5Ac$_2$	2792.4	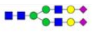	0.447	0.302-0.550	0.467	0.292-0.722	0.273	
27	HexNAc$_5$Hex$_5$Neu5Ac$_1$dHex$_1$	2850.4	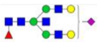	0.005	0.003-0.013	0.002	0.0004-0.011	<0.001	
28	HexNAc$_5$Hex$_6$Neu5Ac$_1$	2880.4	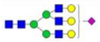	0.016	0.007-0.029	0.009	0.003-0.022	<0.001	
29	HexNAc$_4$Hex$_5$Neu5Ac$_2$dHex$_1$	2966.5	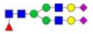	0.011	0.006-0.023	0.015	0.004-0.023	0.106	
30	HexNAc$_5$Hex$_5$Neu5Ac$_2$	3037.5	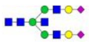	0.002	0.002-0.006	0.002	0.001-0.004	0.002	
31	HexNAc$_5$Hex$_6$Neu5Ac$_1$dHex$_1$	3054.5	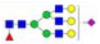	0.002	0.001-0.005	0.001	0.0004-0.003	0.012	
32	HexNAc$_5$Hex$_5$Neu5Ac$_2$dHex$_1$	3211.6	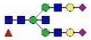	0.001	0.001-0.004	0.001	0.002-0.002	0.071	
33	HexNAc$_5$Hex$_6$Neu5Ac$_2$	3241.6	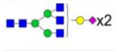	0.016	0.001-0.029	0.016	0.008-0.023	0.604	

34	HexNAc$_5$Hex$_6$Neu5Ac$_2$dHex$_1$	3415.7	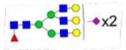	0.002	0.001-0.016	0.006	0.001-0.011	0.001	
35	HexNAc$_5$Hex$_6$Neu5Ac$_3$	3602.8	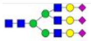	0.052	0.025-0.111	0.067	0.032-0.091	0.016	
36	HexNAc$_6$Hex$_7$Neu5Ac$_2$	3690.8	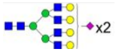	0.006	0.003-0.017	0.006	0.003-0.015	0.708	
37	**HexNAc$_5$Hex$_6$Neu5Ac$_3$dHex$_1$**	3776.9	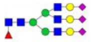	0.011	0.001-0.076	0.056	0.011-0.095	<0.001	0.89 (0.803 - 0.977)
38	HexNAc$_6$Hex$_7$Neu5Ac$_2$dHex$_1$	3864.9	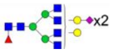	0.001	0.0004-0.013	0.003	0.001-0.008	0.001	
39	**HexNAc$_5$Hex$_6$Neu5Ac$_3$dHex$_2$**	3951.0	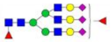	0.001	0.0003-0.006	0.002	0.0004-0.008	0.002	0.75 (0.595 - 0.904)
40	HexNAc$_6$Hex$_7$Neu5Ac$_3$	4052.0	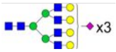	0.008	0.004-0.019	0.013	0.005-0.024	<0.001	
41	**HexNAc$_6$Hex$_7$Neu5Ac$_3$dHex$_1$**	4226.1	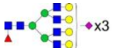	0.001	0.0003-0.015	0.009	0.002-0.017	<0.001	0.88 (0.796 - 0.968)
42	**HexNAc$_6$Hex$_7$Neu5Ac$_3$dHex$_2$**	4400.2	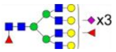	0.001	0.0004-0.006	0.003	0.0004-0.014	<0.001	0.80 (0.654 - 0.939)
43	HexNAc$_6$Hex$_7$Neu5Ac$_4$	4413.2	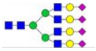	0.010	0.004-0.056	0.026	0.010-0.042	<0.001	
44	**HexNAc$_6$Hex$_7$Neu5Ac$_4$dHex$_1$**	4587.3	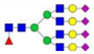	0.002	0.0003-0.025	0.020	0.003-0.051	<0.001	0.91 (0.833 - 0.984)
45	**HexNAc$_6$Hex$_7$Neu5Ac$_4$dHex$_2$**	4761.4	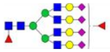	0.001	0.0002-0.015	0.008	0.001-0.047	<0.001	0.87 (0.774 - 0.969)
46	**HexNAc$_6$Hex$_7$Neu5Ac$_4$dHex$_3$**	4935.5	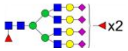	0.0001	0.0002.005	0.002	0.0003-0.023	<0.001	0.79 (0.632 - 0.938)

Table S3. *N*-Glycans (*m/z* < 3600), decreased in primary EOC patients, increased upon intake of chemotherapy. Medians in each cluster of study subjects were calculated from the relative intensities of the *N*-glycans generated from the MALDI-TOF spectra. Min-Max is the minimum and maximum value of the relative intensities of the *N*-glycan peaks in each cluster of study subjects. AUC values evaluated the accuracy of each *N*-glycan in discriminating EOC patients from BOD subjects. AUC values of 0.7 and higher are highlighted in bold; p-values smaller than 0.05 were considered as statistically significant.

***N*-glycan**	**Mass (*m/z*)**	***N*-glycan structure**	**Chemotherapy cycles Relative intensity**			**p-value**	**Chemotherapy cycles AUC**		
			**Pre-treatment Median (min-max)**	**1 - 3 cycles Median (min-max)**	**4 - 6 cycles Median (min-max)**		**Pre-treatment / 1-3**	**1-3 / 4-6**	**Pre-treatment / 4-6**
$HexNAc_3Hex_3$	1416.7	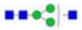	0.001<br>0.0004-0.003	0.002<br>0.0003-0.003	0.002<br>0.001-0.004	0.030	**0.73**	0.55	**0.71**
$HexNAc_2Hex_5$	1579.8	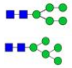	0.013<br>(0.003 -0.020)	0.021<br>(0.005-0.032)	0.022<br>(0.008-0.038)	<0.001	**0.74**	0.60	**0.87**
$HexNAc_3Hex_4$	1620.8	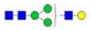	0.001<br>0.0003-0002	0.002<br>0.001-0.003	0.002<br>0.001-0.004	<0.001	**0.81**	0.51	**0.84**
$HexNAc_4Hex_3$	1661.8	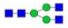	0.001<br>0.0003-0.005	0.003<br>0.001-0.010	0.002<br>0.001-0.006	0.014	**0.75**	0.62	**0.72**
$HexNAc_2Hex_6$	1783.9	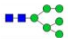	0.013<br>(0.003 -0.020)	0.019<br>(0.005-0.033)	0.021<br>(0.007-0.039)	<0.001	**0.73**	0.59	**0.85**
$HexNAc_3Hex_4Neu5Ac_1$	1982.0	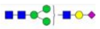	0.004<br>0.001-0.008	0.007<br>0.002-0.011	0.006<br>0.004-0.012	0.005	**0.75**	0.50	**0.79**
$HexNAc_2Hex_7$	1988.0	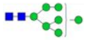	0.004<br>(0.001 -0.009)	0.006<br>(0.002-0.011)	0.006<br>(0.003-0.015)	<0.001	**0.71**	0.65	**0.85**
$HexNAc_4Hex_4dHex_1$	2040.0	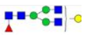	0.009<br>0.002-0.024	0.015<br>0.003-0.026	0.018<br>0.008-0.0.27	0.032	0.63	0.60	**0.74**
$HexNAc_4Hex_5$	2070.0	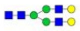	0.003<br>0.001-0.007	0.006<br>0.001-0.010	0.007<br>0.004-0.013	<0.001	**0.75**	0.59	**0.89**
$HexNAc_5Hex_4$	2111.1	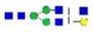	0.002<br>0.001-0.007	0.041<br>0.001-0.009	0.004<br>0.003-0.008	0.041	0.66	0.53	**0.73**

$HexNAc_2Hex_8$	2192.1	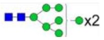	0.008 (0.001 -0.019)	0.012 (0.004-0.020)	0.014 (0.006-0.026)	0.001	0.68	0.67	**0.82**
$HexNAc_4Hex_4Neu5Ac_1$	2227.1	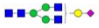	0.007 0.002-0.015	0.011 0.003-0.014	0.010 0.006-0.022	0.044	0.64	0.57	**0.73**
$HexNAc_4Hex_5dHex_1$	2244.1	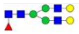	0.002 0.0003-0.006	0.003 0.001-0.008	0.005 0.003-0.008	<0.001	0.67	0.75	**0.86**
$HexNAc_5Hex_4dHex_1$	2285.2	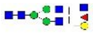	0.001 0.0002-0.002	0.001 0.0003-0.002	0.001 0.0008-0.002	<0.001	**0.74**	0.60	**0.87**
$HexNAc_5Hex_5$	2315.2	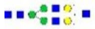	0.001 0.0002-0.002	0.002 0.0003-0.003	0.002 0.001-0.003	0.001	**0.73**	0.54	**0.83**
$HexNAc_3Hex_6Neu5Ac_1$	2390.2	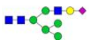	0.003 0.001-0.007	0.004 0.001-0.006	0.005 0.003-0.007	<0.001	**0.70**	0.69	**0.90**
$HexNAc_4Hex_5Neu5Ac_1$	2431.2	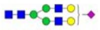	0.090 0.053-0.152	0.105 0.035-0.165	0.129 0.102-0.172	<0.001	0.62	0.73	**0.86**
$HexNAc_5Hex_5dHex_1$	2489.3	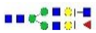	0.001 0.0002-0.003	0.002 0.0004-0.003	0.002 0.001-0.004	0.001	**0.74**	0.53	**0.84**
$HexNAc_5Hex_6$	2519.3	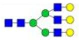	0.001 0.0002-0.002	0.002 0.0004-0.003	0.002 0.001-0.004	<0.001	**0.78**	0.57	**0.88**
$HexNAc_4Hex_5Neu5Ac_1dHex_1$	2605.3	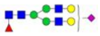	0.006 0.001-0.015	0.007 0.003-0.011	0.009 0.004-0.017	0.019	0.58	0.65	**0.76**
$HexNAc_5Hex_5Neu5Ac_1$	2676.3	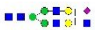	0.004 0.001-0.008	0.005 0.001-0.011	0.007 0.004-0.014	0.017	0.59	0.67	**0.75**
$HexNAc_5Hex_6dHex_1$	2693.4	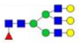	0.001 0.0002-0.002	0.001 0.0003-0.002	0.001 0.001-0.002	<0.001	**0.74**	0.59	**0.86**
$HexNAc_6Hex_6$	2764.4	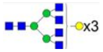	0.003 0.001-0.006	0.004 0.001-0.008	0.005 0.003-0.009	0.001	0.68	0.63	**0.84**
$HexNAc_5Hex_5Neu5Ac_1dHex_1$	2850.4	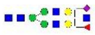	0.002 0.0004-0.011	0.005 0.0008-0.011	0.005 0.003-0.010	0.002	**0.73**	0.55	**0.82**
$HexNAc_5Hex_6Neu5Ac_1$	2880.4	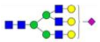	0.009 0.003-0.022	0.014 0.002-0.027	0.017 0.008-0.032	<0.001	**0.72**	0.60	**0.84**

$HexNAc_5Hex_5Neu5Ac_2$	3037.5	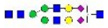	0.002<br>0.001-0.004	0.003<br>0.0004-0.006	0.003<br>0.001-0.004	0.027	0.51	0.65	**0.64**
$HexNAc_4Hex_6Neu5Ac_1dHex_1$	3054.5	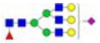	0.001<br>0.0004-0.003	0.002<br>0.001-0.005	0.002<br>0.001-0.003	0.019	**0.70**	0.54	**0.75**

Table S4. *N*-Glycans (*m/z* > 3600), increased in primary EOC patients, decreased upon intake of chemotherapy. Medians in each cluster of study subjects were calculated from the relative peak intensities of the *N*-glycans measured by MALDI-TOF-MS. Min-Max is the minimum and maximum value of the relative intensities of the *N*-glycan peaks in each category of study subjects. AUC values evaluated the accuracy of each *N*-glycan in discriminating EOC patients from BOD subjects. AUC values of 0.7 and higher are highlighted in bold; p-values smaller than 0.05 were considered as statistically significant.

N-glycan	Mass (*m/z*)	*N*-glycan structure	Chemotherapy cycles Relative intensity			p-value	Chemotherapy cycles AUC		
			Pre-treatment Median (min-max)	1 - 3 cycles Median (min-max)	4 - 6 cycles Median (min-max)		Pre-treatment /1-3	1-3 /4-6	Pre-treatment /4-6
$HexNAc_5Hex_6Neu5Ac_2dHex_1$	3415.7	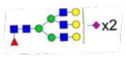	0.006 0.001-0.011	0.005 0.001-0.018	0.003 0.001-0.012	0.023	0.53	**0.71**	**0.73**
$HexNAc_5Hex_6Neu5Ac_3$	3602.8	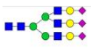	0.067 0.032-0.091	0.060 0.015-0.106	0.077 0.017-0.108	0.391	0.62	0.63	0.54
$HexNAc_5Hex_6Neu5Ac_3dHex_1$	3776.9	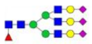	0.056 (0.011 -0.095)	0.035 (0.002-0.088)	0.018 (0.001-0.082)	<0.001	0.68	**0.74**	**0.84**
$HexNAc_6Hex_7Neu5Ac_2dHex_1$	3864.9	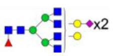	0.003 0.0001-0.008	0.003 0.001-0.019	0.002 0.001-0.008	0.043	0.51	**0.70**	**0.70**
$HexNAc_5Hex_6Neu5Ac_3dHex_2$	3951.0	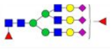	0.002 (0.0004- 0.008)	0.002 (0.001-0.009)	0.001 (0.0003-0.007)	0.008	0.53	**0.77**	**0.74**
$HexNAc_6Hex_7Neu5Ac_3$	4052.0	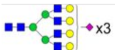	0.013 0.005-0.024	0.014 0.002-0.036	0.012 0.003-0.021	0.818	0.56	0.50	0.54
$HexNAc_6Hex_7Neu5Ac_3dHex_1$	4226.1	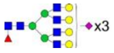	0.009 (0.002-0.017)	0.005 (0.001-0.016)	0.002 (0.0004-0.014)	0.001	0.63	**0.70**	**0.83**
$HexNAc_6Hex_7Neu5Ac_3dHex_2$	4400.2	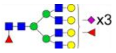	0.003 (0.0004-0.014)	0.002 (0.001-0.009)	0.001 (0.001-0.007)	0.004	0.63	**0.78**	**0.76**
$HexNAc_6Hex_7Neu5Ac_4$	4413.2	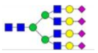	0.026 0.010-0.042	0.018 0.004-0.076	0.018 0.002-0.051	0.071	0.69	0.58	0.68
$HexNAc_6Hex_7Neu5Ac_4dHex_1$	4587.3	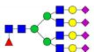	0.020 (0.003-0.051)	0.014 (0.001-0.025)	0.005 (0.0004-0.026)	<0.001	**0.74**	0.66	**0.87**

$HexNAc_6Hex_7Neu5Ac_4dHex_2$	4761.4	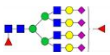	0.008 (0.001-0.047	0.005 (0.0004-0.021)	0.001 0.0003-0.021)	<0.001	0.69	**0.74**	**0.83**
$HexNAc_6Hex_7Neu5Ac_4dHex_3$	4935.5	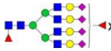	0.002 (0.0003-0.023)	0.001 (0.0003-0.007)	0.0001 (0.0002-0.009)	<0.001	0.60	**0.81**	**0.80**

References

(1) Biskup, K.; Braicu, E. I.; Sehouli, J.; Fotopoulou, C.; Tauber, R.; Berger, M.; Blanchard, V. Serum glycome profiling: a biomarker for diagnosis of ovarian cancer. *Journal of proteome research* **2013**, *12* (9), 4056-4063. DOI: 10.1021/pr400405x

(2) Biskup, K.; Braicu, E. I.; Sehouli, J.; Tauber, R.; Blanchard, V. The serum glycome to discriminate between early-stage epithelial ovarian cancer and benign ovarian diseases. *Disease markers* **2014**, *2014*, 238197. DOI: 10.1155/2014/238197
